# Circulating adipocyte fatty acid-binding protein exacerbates LPS-induced neurotoxicity by crossing the disrupted blood–brain barrier and promoting neuronal apoptosis

**DOI:** 10.1186/s12964-026-02680-y

**Published:** 2026-01-23

**Authors:** Muhammad Mustapha Ibrahim, Chunyan Li, Linhui Qiu, Yue Hu, Aimin Xu, Shilun Yang, Junlei Chang, Cheng Fang

**Affiliations:** 1 State Key Laboratory of Biomedical Imaging Science and System, Guangdong-Hong Kong Joint Laboratory for Metabolic Medicine, Institute of Biomedicine and Biotechnology, Shenzhen Institutes of Advanced Technology, Chinese Academy of Sciences, Xueyuan Avenue 1068, Nanshan, Shenzhen, Guangdong 518055 China; 2 University of Chinese Academy of Sciences, Beijing, China; 3 Department of Biomedical Engineering, Southern University of Science and Technology, Shenzhen, Guangdong China; 4 Department of Pharmacology and Pharmacy, LKS Faculty of Medicine, The University of Hong Kong, Pokfulam, Hong Kong China; 5 State Key Laboratory of Pharmacological Biotechnology, LKS Faculty of Medicine, The University of Hong Kong, Pokfulam, Hong Kong China; 6 Stroke Center, Department of Neurology, The First Hospital of Jilin University, Changchun, China; 7 Neuroscience Research Center, Department of Neurology, The First Hospital of Jilin University, Changchun, Jilin China

**Keywords:** A-FABP, Sepsis-associated encephalopathy, Blood‒brain barrier, Neuroinflammation, LPS, Neuronal apoptosis

## Abstract

Sepsis-associated encephalopathy (SAE) is a critical complication of systemic inflammation with poorly understood mechanisms. This study identified adipocyte fatty acid-binding protein (A-FABP or FABP4) as a key mediator linking peripheral inflammation to central nervous system (CNS) damage. Using an LPS-induced endotoxemia model in wild-type and *Fabp4* knockout (KO) mice, we demonstrated that circulating A-FABP (1) crosses the compromised blood‒brain barrier (BBB), (2) accumulates in hippocampal neurons, and (3) synergizes with LPS to drive neuronal apoptosis. The monoclonal antibody 6H2, which neutralizes A-FABP, significantly alleviated BBB dysfunction, attenuated neuroinflammation, and improved neuronal survival. In vitro studies confirmed that HT22 neurons internalize exogenous A-FABP, which amplifies LPS-induced late apoptosis without affecting early apoptotic pathways. These findings establish circulating A-FABP as both a biomarker and therapeutic target for SAE, revealing a novel periphery-to-CNS inflammatory cascade.

**Supplementary Information:**

The online version contains supplementary material available at 10.1186/s12964-026-02680-y.

## Introduction

Sepsis-associated encephalopathy (SAE) is a frequent and severe complication of systemic inflammation, characterized by blood–brain barrier (BBB) disruption, neuroinflammation, and neuronal dysfunction [1–3]. Despite its prevalence, the mechanisms linking peripheral inflammation to central nervous system (CNS) injury remain poorly defined, and effective therapies are lacking [4–6]. BBB breakdown facilitates the entry of circulating inflammatory mediators into the brain, triggering microglial activation and cytokine release, which contribute to cognitive impairment and increased mortality in sepsis patients [7–13]. Identifying specific molecular drivers that cross the compromised BBB and exacerbate neuronal damage is critical for developing targeted neuroprotective strategies.

Adipocyte fatty acid-binding protein (A-FABP), a lipid chaperone expressed in adipocytes and macrophages, regulates systemic metabolism and inflammation [14, 15]. It has been implicated in insulin resistance, metabolic syndrome, and cardiovascular diseases, and recent studies suggest a role in sepsis pathogenesis [16–24]. Studies have shown that A-FABP inhibition, either genetically or pharmacologically, mitigates septic acute kidney injury by disrupting the TLR4/c-Jun signaling pathway, which increases inflammation and cell death [16]. Higher A-FABP levels correlate with increased mortality both in sepsis mice and patient, suggesting its role as a mediator of sepsis severity and a potential biomarker for poor outcomes [25–27]. A-FABP may bridge metabolic and inflammatory pathways in neurological disorders. In macrophages and microglia, A-FABP potentiates TLR4 signaling, the same pathway activated by LPS [28, 29]. But whether A-FABP actively contributes to CNS injury or merely reflects disease severity remains unexplored. This knowledge gap is significant because A-FABP neutralization improves outcomes in metabolic diseases, raising the possibility of repurposing this approach for neuroprotection.

Our study firstly evaluated the therapeutic potential of 6H2, a potent and specific A-FABP neutralizing monoclonal antibody (mAb) we developed previously [21], in preserving BBB integrity and reducing neuroinflammation in a murine sepsis model. Then, we addressed three fundamental questions: First, does circulating A-FABP infiltrate the brain during systemic inflammation? Second, what cellular populations internalize A-FABP? Third, does A-FABP interact with LPS to exacerbate neuronal injury? Using complementary in vivo (*Fabp4* KO mice) and in vitro (HT22 neurons) models, we revealed a novel periphery-to-CNS inflammatory cascade and establish A-FABP as both a biomarker and therapeutic target for SAE.

## Methods

### Animals and treatments

8-week-old male C57BL/6J mice were bought from the Vital River Laboratory in Beijing, China. The mice were kept in a clean, pathogen-free environment with a standard 12-hour light and dark cycle. They had free access to food and water throughout the study. All animal handling and experimental procedures complied with the NIH guidelines for ethical animal care (NIH Publication No. 86 − 23, revised 1985) and were approved by the Institutional Animal Care and Use Committee (IACUC) at the Shenzhen Institute of Advanced Technology, Chinese Academy of Sciences.

To induce endotoxemia, LPS (*Escherichia coli* O55:B5, Sigma, Cat. No. L2880) was injected into the abdominal cavity at a dose of 25 mg/kg body weight. 30 min after the LPS injection, the mice received an intravenous dose of either the mouse mAb 6H2 (3.6 mg/kg) or a control antibody (mouse IgG, Immuno-Diagnostics, Cat. No. 221116) at the same concentration. Any mice that died before the scheduled endpoint were excluded from the analysis. The assignment of the mice to treatment groups was randomized to ensure unbiased results.

The constitutive *Fabp4* KO mouse strain was generously provided by Dr. Ruby L-C Hoo (LKS Faculty of Medicine, The University of Hong Kong) [30]. In this study, we used 8-week-old male A-FABP KO mice alongside their wild-type (WT) littermate controls.

### Cell culture and treatments

The murine hippocampal neuronal cell line HT22 (Merck, #SCC129) and the microglial cell line BV2 (Procell, #CL-0493) were cultured in Dulbecco’s modified Eagle’s medium (DMEM; Cytiva, #sh30243.01) supplemented with 10% fetal bovine serum (FBS; Gibco, #10270106) and 1% penicillin/streptomycin (Gibco, #15140122) at 37 °C in a humidified 5% CO₂ incubator. For the experimental treatments, the cells were serum starved (HT22, 24 h; BV2, 12 h) and exposed to the indicated concentrations of lipopolysaccharide (LPS; Sigma, #L2880) or recombinant murine A-FABP (MCE, #HY-P75215). Treatments were administered for the indicated time in complete medium. The control groups received vehicle (PBS) only.

### ELISA

Serum levels of A-FABP were quantified using a commercial sandwich ELISA kit (Mouse A-FABP ELISA Kit, ImmunoDiagnostics, #32030) according to the manufacturer’s instructions. Briefly, serum samples were diluted 1:200 in the provided sample diluent. Standards and diluted samples were added to antibody-precoated wells and incubated for 2 h at room temperature. After washing, a biotinylated detection antibody was added and incubated for 1 h, followed by streptavidin-HRP conjugate incubation for 30 min. After final washes, the reaction was developed with TMB substrate, stopped with 2 M H₂SO₄, and the absorbance was immediately measured at 450 nm using a microplate reader (Thermo Scientific, Multiskan GO). All samples and standards were assayed in duplicate. The concentration of A-FABP in each sample was interpolated from the standard curve.

### Measurement of Evans blue extravasated into the brain

Four hours before sacrifice, anesthetized mice received an injection of Evans blue (EB) (2%, 4 mL/kg) via the tail vein. After anesthesia, the mice were perfused transcardially with 40 mL of ice-cold PBS to flush out any remaining dye in the bloodstream. The brain was then carefully removed and homogenized in 1 mL of 50% trichloroacetic acid. The homogenate was centrifuged at 12,000 rpm for 15 min, and the EB concentration in the supernatant was measured via a spectrophotometer (Thermo Fisher, Multiskan GO) at 620 nm. A standard curve was used to calculate the EB levels, which were expressed as ng per gram of brain tissue.

### Immunofluorescence staining

For tissue processing, deeply anesthetized mice were transcardially perfused with cold PBS, followed by brain extraction. The harvested tissues (2 mm-thick sections) were fixed in 4% formalin for 1 h at room temperature and then cryoprotected by sequential dehydration in 15% and 30% sucrose solutions at 4 °C for 24 h. After being embedded in OCT compound (Sakura, Cat. #4583), the tissues were sectioned into 20 μm-thick slices, air-dried, and washed with PBS. For immunostaining, the sections were subjected to acetone fixation at −20 °C for 30 min, SDS-mediated antigen retrieval for 30 min at room temperature, and blocking with a solution containing 10% donkey serum, 1% BSA, 1% Triton X-100, and 0.05% NaN3. Primary and secondary antibodies (Tables S1-S2) were applied overnight at 4 °C or for 1 h at RT, respectively. The slides were mounted with ProLong Gold Antifade Reagent with DAPI (Cell Signaling Technology, Cat. #8961S).

Fluorescence imaging was performed via a ZEISS Axio Imager Z2 microscope with ApoTome.2. Quantitative analysis included assessment of BBB integrity (target protein density/CD31 area), microglial activation (IBA1^+^ cells/area, endpoints per cell), and astrocyte reactivity (GFAP mean density), all of which were performed via ImageJ software. The hippocampus and cortex were specifically analyzed for these parameters to evaluate neurovascular and neuroinflammatory responses.

### RNA extraction and RT‒qPCR analysis

The mice were deeply anesthetized and transcardially perfused with ice-cold PBS. The brain tissue was carefully dissected on ice and homogenized in RNA lysis buffer. For cultured cells, the medium was aspirated, and the cells were rinsed once with PBS. Total RNA was isolated via the FastPure Cell/Tissue Total RNA Isolation Kit V2 (Vazyme), followed by cDNA synthesis with HiScript III RT SuperMix for qPCR (+ gDNA wiper) (Vazyme) to ensure genomic DNA removal.

For gene expression analysis, RT‒qPCR was conducted on a LightCycler 96 system (Roche) with the following thermal profile: initial denaturation at 95 °C for 3 min, followed by 40 cycles of 95 °C for 15 s and 60 °C for 1 min. We quantified the expression levels of inflammatory markers (*Tnf*, *Il1b*, *Il6*, *Il17a*, *Ccl2*, and *Fabp4*) via the use of β-actin as a housekeeping gene. All primers (Table S1) were designed with a 60 °C annealing temperature and were verified via melt curve analysis. Relative gene expression was determined via normalization to β-actin via the ΔΔCt method.

### Western blot analysis

Brain tissue was homogenized in RIPA lysis buffer (Solarbio, #R0010) supplemented with Complete Protease Inhibitor Cocktail and PhosStop phosphatase inhibitor tablets (Roche, #11697498001 and #04906837001). After homogenization, the lysates were centrifuged at 14,000 × g for 15 min at 4 °C to pellet tissue debris. The resulting supernatant was collected as the protein extract. For cultured cells, protein lysates were prepared by adding an appropriate volume of the same RIPA lysis buffer. Protein concentration was determined using a BCA protein assay kit (Solarbio, #BC1720) according to the manufacturer’s protocol. Protein samples (10–40 µg per lane) were resolved via 12% SDS‒PAGE and subsequently transferred to 0.22 μm PVDF membranes. Following transfer, the membranes were blocked with 5% nonfat milk in TBST for 1 h at room temperature to prevent nonspecific binding. The following primary antibodies were used: Polyclonal Goat anti-FABP4/A-FABP Antibody (R&D system, Cat. #AF1443), Beta Actin Monoclonal antibody (Proteintech, Cat. #66009-1-Ig).

After extensive washing, the membranes were incubated with the following secondary antibodies: HRP-linked Anti-mouse IgG Antibody (Cell Signaling, Cat. #7076), HRP-conjugated Rabbit Anti-Goat IgG (Proteintech, Cat. #SA00001-4). Following additional TBST washes, protein bands were visualized via a chemiluminescent HRP substrate (Millipore, Cat. #WBKLS0500) and imaged via the GelView 6000 M system (BioLight, GV6000). Quantitative analysis was performed via ImageJ software, with target protein expression levels normalized to those of β-actin or GAPDH as loading controls.

### Flow cytometric analysis of immune cells in the brain

The mouse brain tissue was minced into fine pieces using a sterilized razor blade. The minced tissue was then broken down using an enzyme cocktail containing Type IV collagenase (400 U/ml, Worthington, Cat. #LS004186), Dispase (1.2 U/ml, Worthington, Cat. #LS02100), and DNase I (32 U/ml, Worthington, Cat. #LS006333), dissolved in 6 mL of DPBS with Mg^2+^/Ca^2+^ (one mouse). This digestion process ran for 30 min at 37 °C, with gentle mixing every 10 min to help break the tissue.

Following digestion, 1 mL of FBS and 20 mL of chilled PBS was added to stop the reaction and then passed the mixture through 40 μm filters. The cells were spun at 400 × g for 5 min at 4 °C; then, they were resuspended in 20% BSA solution for myelin removal at 1,000 × g (25 min, 4 °C). The cells were washed twice in 0.3% FBS/PBS solution with centrifugation at 400× g each time.

For surface marker analysis, the cells were first incubated with a mouse BD Fc block in 0.3% FBS/PBS for 10 min on ice to prevent nonspecific antibody binding. After being washed with PBS, the cells were stained with anti-CD11b-APC (BioLegend, Cat. #101211) and PE-conjugated anti-CD45 (BD Biosciences, Cat. #516087) for 45 min at 4 °C in the dark. The stained cells were washed and resuspended in PBS for acquisition. Flow cytometric analysis was performed via a Sony MA900 flow cytometer equipped with a 100 μm nozzle, and CD45⁺/CD11b⁺ populations were identified for downstream analysis.

### Cell apoptosis assay

The cells were digested with 0.25% trypsin (Gibco, Cat. #15050065) for 1 min at 37 °C. The suspension was subsequently centrifuged at 300 × g for 5 min at 4 °C. The cells were stained with the Annexin V-PE/7-AAD Apoptosis Detection Kit (Yeasen, Cat. #40310ES50) following the manufacturer’s protocol. Briefly, after treatment, the cells were washed twice with cold PBS and resuspended in Annexin V binding buffer. Annexin V-PE and 7-AAD were then added to the cell suspension and incubated for 15 min at room temperature in the dark. The samples were analyzed via flow cytometry (Beckman, CytoFLEX S) within one hour of staining. Viable, early apoptotic, and late apoptotic cells were distinguished at the basis of their staining profiles: Annexin V⁻/7-AAD⁻ (viable), Annexin V⁺/7-AAD⁻ (early apoptotic), and Annexin V⁺/7-AAD⁺ (late apoptotic). All flow cytometric data were analyzed via FlowJo software.

### Murine sepsis score development

In the LPS-induced sepsis model, disease progression and treatment effects were monitored using a refined behavioral assessment [31]. Following baseline observations, the animals were scored at regular intervals via a murine sepsis score (MSS) adapted for the LPS model. The MSS comprises six parameters, spontaneous movement, stimulus response, posture, respiratory rate, breathing pattern, and piloerection, each graded from 0 (normal) to 4 (severely impaired), yielding a total score indicative of illness severity. Data were recorded on standardized sheets alongside rectal temperature and body weight measurements. The MSS provides a quantitative, reproducible framework for tracking sepsis severity, enabling comparisons across the sham, IgG-treated, and 6H2-treated groups. Validation against physiological markers, including hypothermia and weight loss, confirmed the system’s sensitivity and alignment with established models.

### Cell viability assay (CCK-8)

HT22 cells were seeded into 96-well plates at a density of 5,000 cells/well in 100 µL of complete DMEM and incubated for 24 h at 37 °C in a humidified 5% CO₂ atmosphere to allow cell attachment. Following attachment, the cells were treated with lipopolysaccharide (LPS), recombinant A-FABP, or vehicle controls in complete medium for 0, 6, 24, or 48 h. After treatment, 10 µL of Cell Counting Kit-8 (Yeasen, Cat. #40203ES80) solution was added to each well and incubated for an additional 1 h at 37 °C. The absorbance was then measured at 450 nm via a microplate reader (Thermo Scientific, Multiskan GO).

### Quantitative analysis

All quantitative data are presented as the mean ± standard error of the mean (SEM). Statistical analyses were performed via GraphPad Prism version 10 (GraphPad Software, CA, USA). For comparisons between two groups, unpaired two-tailed Student’s *t* tests were applied, whereas one-way analysis of variance (ANOVA) was used for comparisons involving more than two groups. A *p* value less than 0.05 was considered statistically significant.

For the analysis of microglial morphology, an ImageJ-based skeletonization protocol was employed [32]. Briefly, original fluorescence micrographs were preprocessed via fast Fourier transform (FFT) bandpass filtering and unsharp masking to increase image quality. The processed images were then binarized and skeletonized, followed by quantification of microglial branching structures via the Analyze Skeleton plugin. Cropped overlays were used to ensure the anatomical accuracy of segmentation. Endpoint counts derived from skeletons were normalized to the number of microglial somata per field to account for cellular density.

In this study, 158 mice were enrolled across all experiments, 37 mice died prior to reaching the predefined study endpoints; these losses occurred exclusively within the LPS-treated groups and were distributed across experiments. Thus, 113 mice were included in the final analyses. The observed variation in group sizes (e.g., 4/6/6 or 4/7/7) reflects these deaths (Table S2).

## Results

### The A-FABP-neutralizing antibody 6H2 rescued survival and reduced disease severity in LPS-induced endotoxemia

**Fig. 1:**
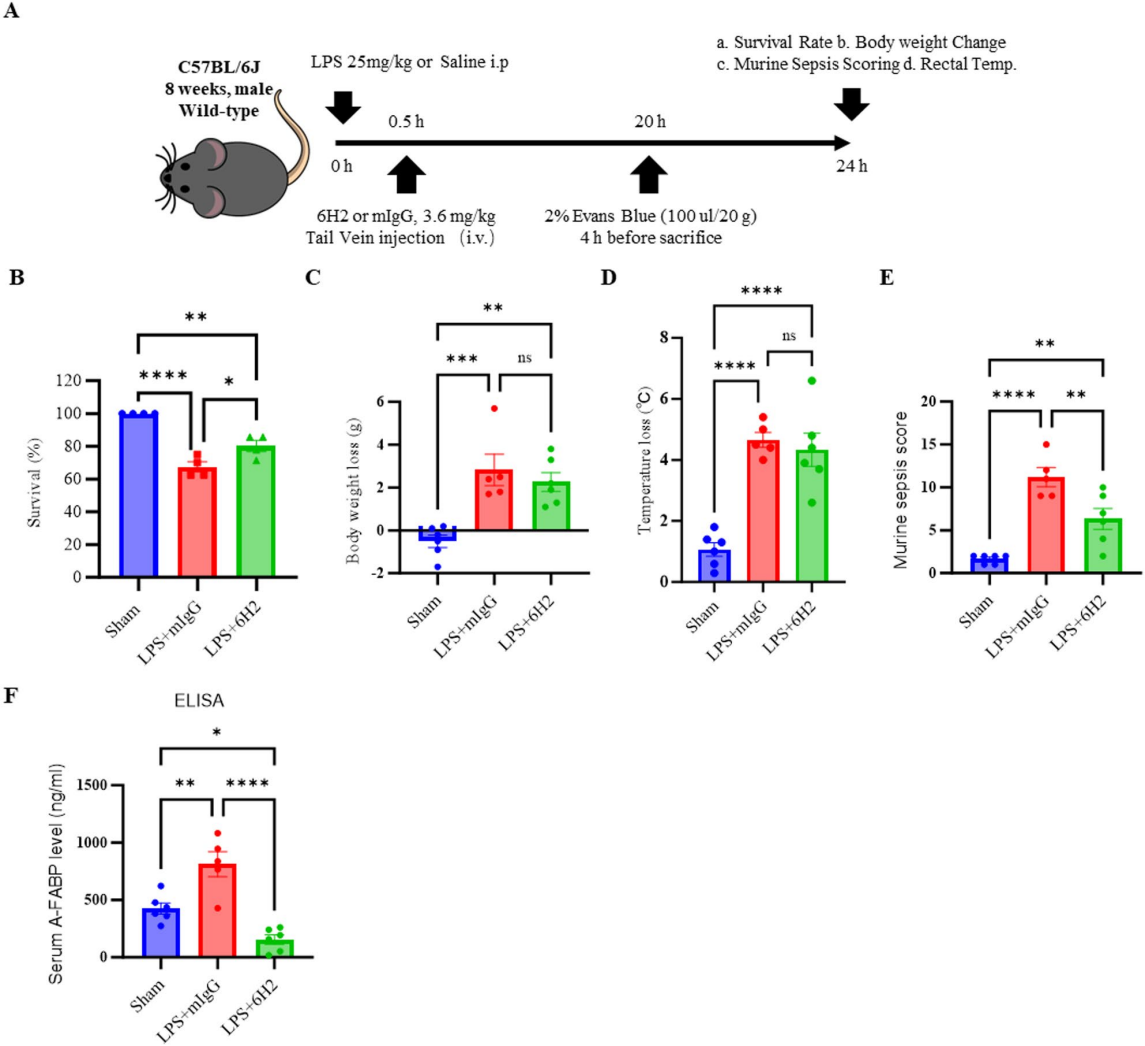
The A-FABP-neutralizing antibody 6H2 rescues survival and reduces disease severity in LPS-induced endotoxemia. **A** Schematic of the experimental timeline. Mice received an intraperitoneal injection of LPS (25 mg/kg) followed by intravenous administration of 6H2 (3.6 mg/kg) or control mouse IgG (mIgG) 30 min later. Endpoints were assessed at 24 h. **B** Survival rate over 24 h (data was from for independent experiments, each dot represented one experiment, Table S2). **C**, **D** Change in body weight (**C**) and core temperature (**D**) at 24 h post-LPS. **E** Murine Sepsis Score (MSS) at 24 h post-LPS. **F** Serum A-FABP levels measured by ELISA at 24 h post-LPS. Data in (**C**-**F**) were from animals surviving to the 24-hour endpoint (Sham, *n* = 6; LPS + mIgG, *n* = 5; LPS + 6H2, *n* = 6) and presented as mean ± SEM. Statistical significance was determined by one-way ANOVA with Tukey’s post hoc test (*p < 0.05, **p < 0.01, ***p < 0.001, ****p < 0.0001; ns, not significant)

In this study, 8-week-old male C57BL/6J wild-type mice were used to establish an LPS-induced (25 mg/kg body weight) sepsis model to evaluate the therapeutic effects of the monoclonal antibody 6H2. The experimental groups included a saline control group (Sham), an LPS + mIgG control group (LPS + mIgG), and an LPS + 6H2 treatment group (LPS + 6H2). 30 min after intraperitoneal LPS injection, the mice received either mIgG or 6H2 (3.6 mg/kg) via tail vein injection. Survival rates, body weight changes, body temperature fluctuations, and sepsis scores were assessed 24 h later (Fig. 1A). The results revealed survival rates of 100% in the control group, 67.5% in the mIgG group, and 80% in the 6H2 group (Fig. 1B). All septic mice exhibited significant weight loss, hypothermia, and motor dysfunction (manifested as reduced spontaneous movement, jerky motions, abnormal gait, piloerection, partially closed eyelids, and ocular discharge) within 24 h. Compared with the mIgG controls, 6H2 treatment failed to reverse the LPS-induced metabolic disturbances reflected by persistent weight loss and hypothermia (Fig. 1C and D). Sepsis scoring confirmed minimal septic manifestations in the sham group, severe sepsis in the LPS + mIgG group, and significant symptom alleviation in the LPS + 6H2 group (Fig. 1E). Notably, compared with septic control mice, 6H2-treated mice presented reduced neurological manifestations, including less piloerection, improved eye opening, and decreased ocular discharge, suggesting specific neuroprotective effects independent of systemic metabolic recovery.

Consistent with clinical observations in septic patients, LPS challenge induced a significant, approximately two-fold increase in circulating A-FABP levels. This elevation was completely abrogated by 6H2 treatment, which reduced serum A-FABP to levels significantly below baseline (Fig. 1F). This result confirms the translational relevance of our model and provides direct in vivo evidence of rapid and potent target engagement by the 6H2 antibody.

Collectively, these findings demonstrate that 6H2 treatment improves survival, mitigates clinical sepsis severity, and effectively neutralizes the rise in circulating A-FABP, highlighting its therapeutic potential for modulating the dysregulated immune response in endotoxemia.

### 6H2 treatment alleviated LPS-induced blood‒brain barrier disruption

**Fig. 2:**
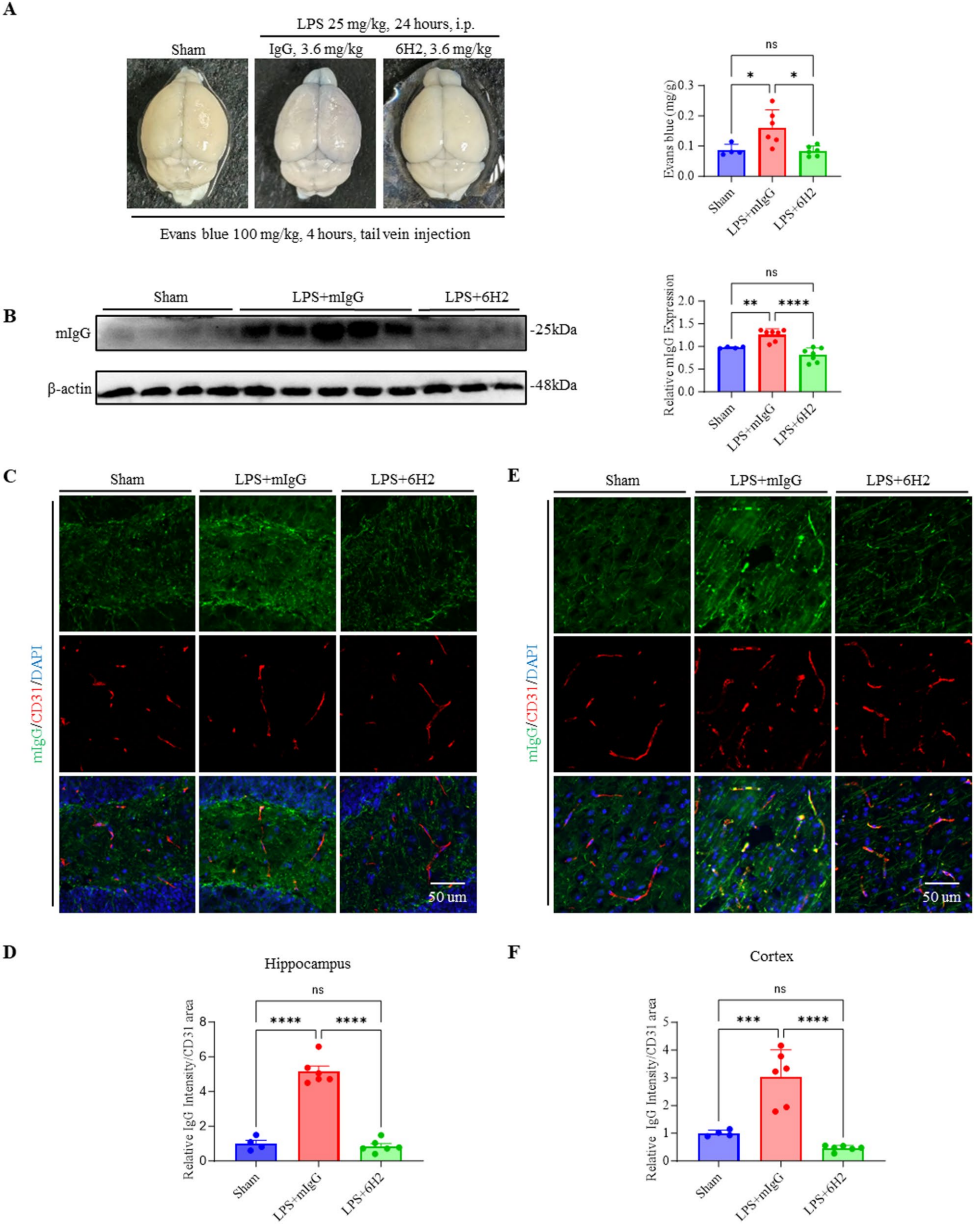
6H2 treatment preserves blood–brain barrier integrity during endotoxemia. **A** Representative images of mouse brain and quantification of Evans Blue extravasation in brain tissue. **B** Representative Western blot and quantification of IgG levels in brain homogenates. (Sham, *n* = 6; LPS + mIgG, *n* = 7; LPS + 6H2, *n* = 7). **C**–**F** Immunofluorescence images and quantification revealed that 6H2 reduces IgG extravasation normalized with CD31^+^ vasculature area of the hippocampal (**C**-**D**) and cortical (**E**-**F**) regions. Scale bars: 50 μm. Data in (**A**, **C**-**F**) were from animals surviving to the 24-hour endpoint (Sham, *n* = 4; LPS + mIgG, *n* = 6; LPS + 6H2, *n* = 6). The data were presented as the mean ± SEM. Statistical significance was determined by one-way ANOVA with Tukey’s post hoc test (*p < 0.05, **p < 0.01, ***p < 0.001, ****p < 0.0001; ns, not significant)

LPS-induced sepsis caused significant BBB leakage, neuroinflammation, and systemic inflammation, leading to severe neurological damage [3, 33–36]. To evaluate the ability of 6H2 to protect against LPS-induced blood‒brain barrier (BBB) disruption, we employed complementary approaches. Exogenous Evans blue extravasation and endogenous IgG infiltration were used to quantify BBB integrity. LPS-challenged mice presented increased Evans blue penetration into the brain parenchyma, and 6H2 treatment significantly attenuated Evans blue extravasation (Fig. 2A). Western blot analysis further supported this finding, as the LPS + mIgG group presented elevated IgG levels in brain tissue, whereas the LPS + 6H2 group presented reduced mIgG levels (Fig. 2B). Additionally, co-immunofluorescence staining of plasma IgG and CD31 (endothelial cell markers) in the hippocampus and cortex revealed intense IgG staining in the LPS + mIgG group, whereas the LPS + 6H2 group presented significantly reduced IgG staining, further supporting the protective effects of 6H2 on BBB integrity (Fig. 2C and D). These complementary approaches consistently demonstrated that 6H2 preserves BBB integrity during sepsis.

### 6H2 treatment alleviated LPS-induced neuroinflammation

**Fig. 3:**
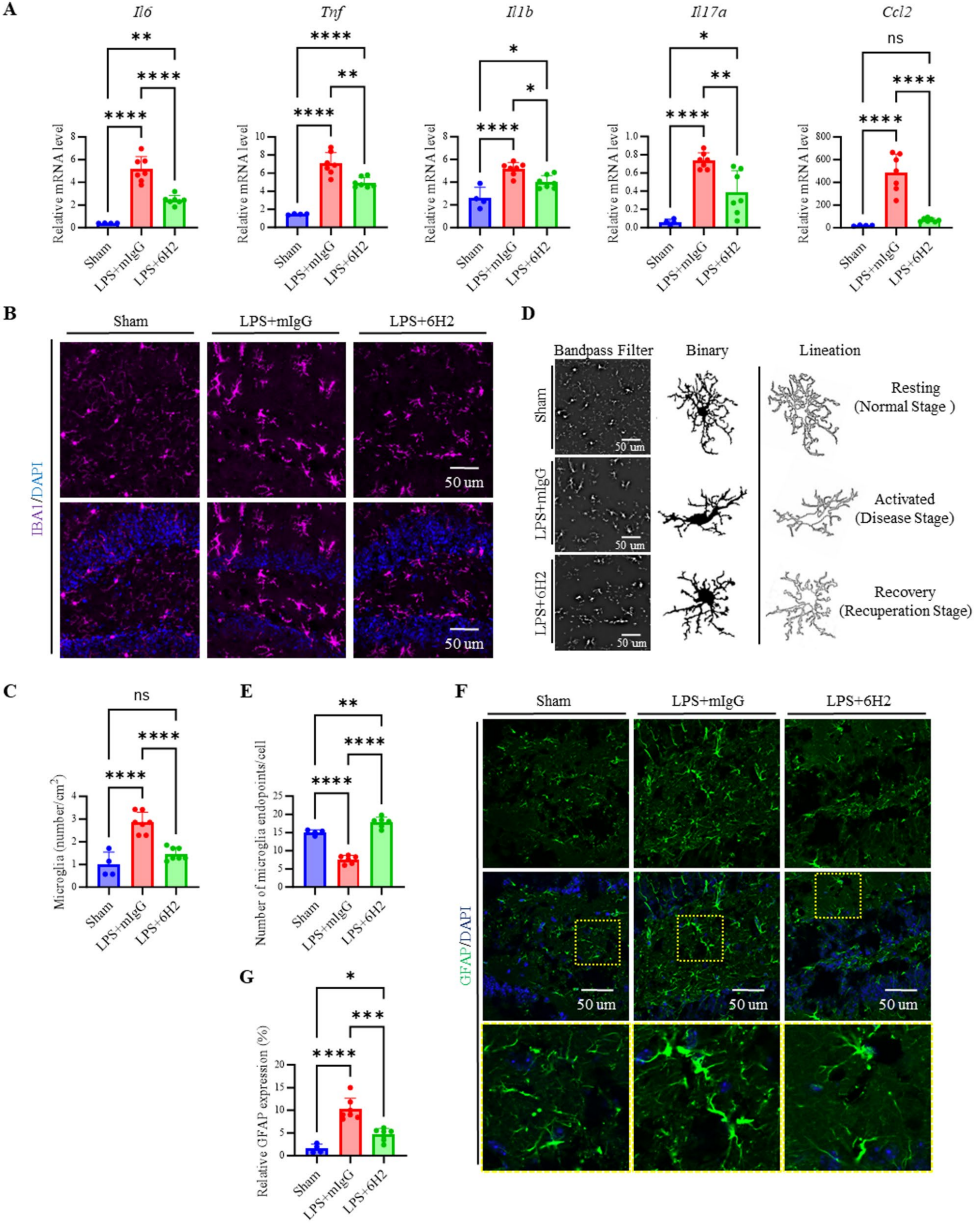
6H2 treatment reduces neuroinflammation and suppresses glial activation in LPS-induced endotoxemia. **A** RT-qPCR analysis of pro-inflammatory cytokine and chemokine mRNA levels in the brain homogenate. **B-C** Analysis of microglial response. **B**-**C** Representative images of IBA1⁺ microglia (**B**) and quantification of IBA^+^ cell number per cm^2^ (**C**) in the hippocampus. Scale bar: 50 μm. **D**-**E** Representative images of endpoints of microglia (**D**) and quantification of microglial morphological complexity, expressed as endpoints per cell (**E**). **F**-**G** Analysis of astrocytic response. Representative images of GFAP⁺ astrocytes in the hippocampus (**F**) and quantification of GFAP mean fluorescence intensity (**G**). Scale bar: 50 μm. Data were from animals surviving to the 24-hour endpoint (Sham, *n* = 4; LPS + mIgG, *n* = 7; LPS + 6H2, *n* = 7) and were presented as mean ± SEM. Statistical significance was determined by one-way ANOVA with Tukey’s post-hoc test (*p < 0.05, **p < 0.01, ***p < 0.001, ****p < 0.0001; ns, not significant)

LPS-induced sepsis elicited both vascular and neuroinflammatory responses, as evidenced by the upregulation of proinflammatory cytokines and microglial and astrocyte activation [3]. Next, we investigated whether 6H2 treatment could mitigate neuroinflammation. RT‒qPCR analysis revealed that LPS significantly increased the expression of proinflammatory cytokines (*Tnf*, *Il1b*, *Il6*, *Il17a*, and *Ccl2*) in the brain (Fig. 3A), but 6H2 treatment notably reduced their expression, resulting in cytokine levels closer to those observed in the Sham group. Immunofluorescence staining of IBA1, a microglial marker, in the hippocampus revealed that LPS-treated mice exhibited significant microglial activation, which was notably reduced by 6H2 treatment (Fig. 3B-C). Microglial morphology varied among the groups: a resting phenotype in the Sham group (~ 15 Endpoints/Cell), an activated phenotype in the IgG-treated group (~ 7 Endpoints/Cell, *p* < 0.0001 vs. Sham), and a recuperative phenotype in the 6H2-treated group (~ 17 Endpoints/Cell, *p* < 0.0001 vs. IgG). Quantitative analysis confirmed that 6H2 effectively diminished LPS-induced microglial activation (Fig. 3D-E). Similarly, LPS-induced astrocyte activation is characterized by morphological changes, including increased cell size, complexity, and process density. These changes are indicative of a reactive state in astrocytes [37]. GFAP staining, an astrocyte marker, revealed that 6H2 alleviated LPS-induced astrocytic activation, as characterized by increased size, complexity, and process density in the hippocampus (Fig. 3F-G). Collectively, these findings suggest that 6H2 mitigates LPS-induced neuroinflammation by reducing proinflammatory cytokine expression and suppressing the activation of both microglia and astrocytes.

### 6H2 treatment improved neuronal survival in LPS-induced sepsis

**Fig. 4:**
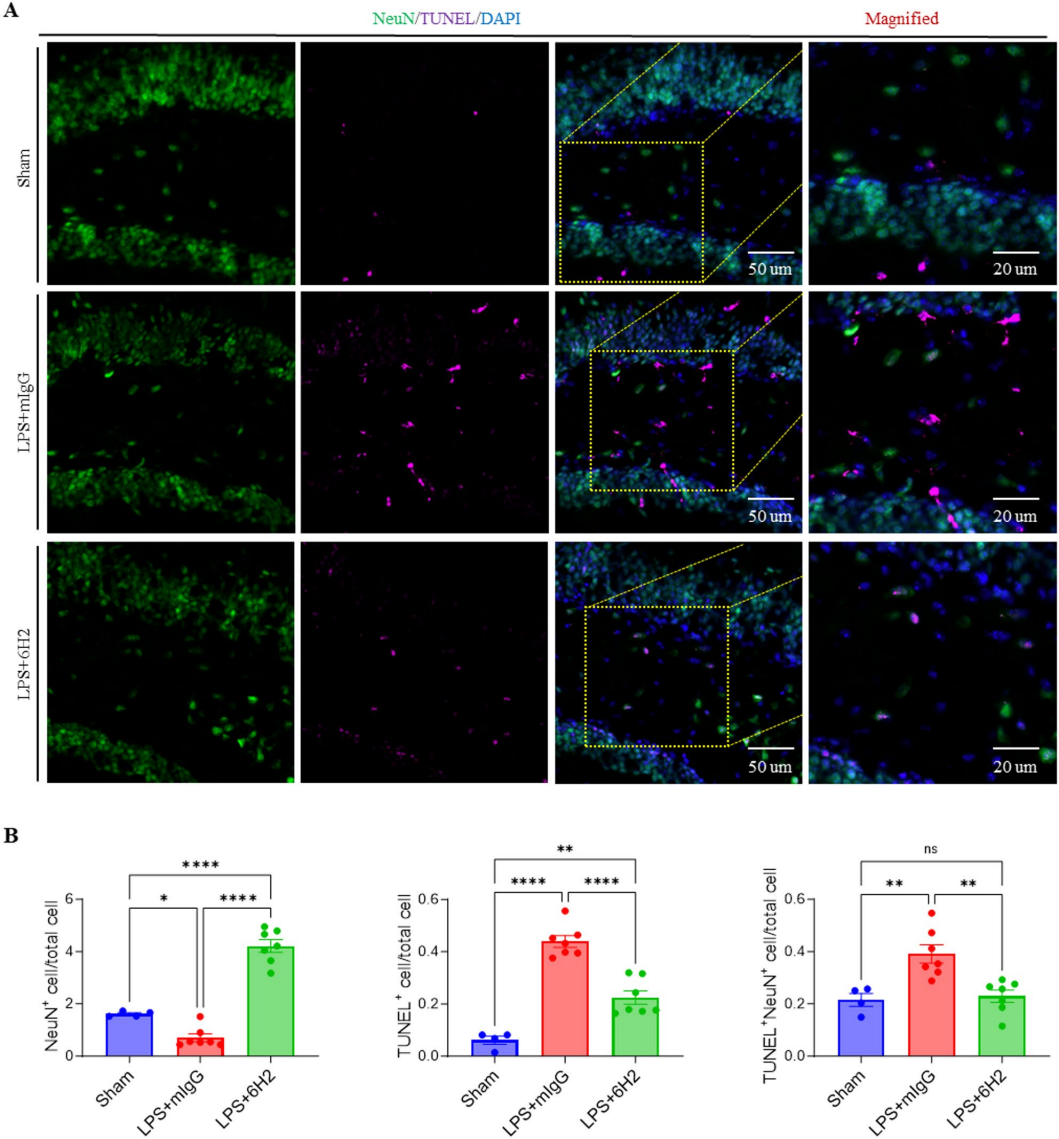
6H2 treatment attenuates LPS-induced neuronal apoptosis in the hippocampus. **A** Representative immunofluorescence images of hippocampal sections stained for the neuronal marker NeuN (green) and the apoptotic cells by TUNEL assay (magenta). Insets showed higher-magnification views of the boxed areas. Scale bars: 50 μm (overview), 20 μm (insets). **B** Quantification of NeuN^+^ cell number normalized with total cell number (left), TUNEL⁺ cell number normalized with total cell number (middle), and NeuN^+^TUNEL^+^ cell number normalized with total cell number (right) in the hippocampal region. Data were from animals surviving to the 24-hour endpoint (Sham, *n* = 4; LPS + mIgG, *n* = 7; LPS + 6H2, *n* = 7) and were presented as mean ± SEM. Statistical significance was determined by one-way ANOVA with Tukey’s post-hoc test (*p < 0.05, **p < 0.01, ***p < 0.001, ****p < 0.0001; ns, not significant)

Given that 6H2 preserves BBB integrity and attenuates neuroinflammatory responses, we next investigated its neuroprotective potential upon LPS administration. To assess neuronal apoptosis, we conducted the immunofluorescence staining for NeuN (a neuronal marker) and subsequently performed a terminal deoxynucleotidyl transferase-mediated dUTP nick end labeling (TUNEL) assay in hippocampal tissue **(**Fig. 4A**)**. Immunohistochemical analysis revealed significant differences in neuronal survival and apoptosis across the experimental groups **(**Fig. 4B**)**. Compared with the Sham group, which presented abundant NeuN^+^ cells and minimal TUNEL^+^ staining, indicating healthy neuronal populations with negligible apoptosis, the LPS + mIgG group presented severe neuronal damage characterized by a marked reduction in NeuN^+^ cells and a substantial increase in TUNEL^+^ cells. This pattern demonstrated extensive LPS-induced apoptosis of the brain cells. Importantly, the LPS + 6H2 treatment group showed significant protection of the brain cells, with preserved NeuN^+^ cell counts and dramatically fewer TUNEL^+^ cells than the LPS + mIgG group. Further analysis of NeuN^+^TUNEL^+^ double-labeled cells confirmed these findings, whereas the Sham group presented virtually no apoptotic neurons, the LPS + mIgG group presented numerous double-positive cells, and this effect was significantly attenuated by 6H2 treatment. These results clearly demonstrate that 6H2 confers robust neuroprotection in the LPS-induced sepsis model by maintaining neuronal viability and reducing apoptosis, suggesting its potential therapeutic value for sepsis-associated neurological damage.

To assess a more clinically relevant treatment window, we administered 6H2 or control IgG 3 h after LPS challenge and evaluated outcomes at 24 h **(**Fig. S2A). While delayed 6H2 treatment did not significantly reverse LPS-induced systemic symptoms (weight loss, hypothermia), it reduced the Murine Sepsis Score (Fig. S2B–D). Importantly, it still significantly protected the CNS, attenuating blood-brain barrier disruption (Evans Blue and IgG extravasation) and reducing hippocampal neuronal apoptosis (Fig. S2E–G). In summary, these findings demonstrate that 6H2 retains substantial neuroprotective and BBB-stabilizing efficacy even when administered at a delayed, more clinically relevant time point. This defines a promising therapeutic window for A-FABP neutralization and strengthens its potential as a translatable strategy for sepsis-associated encephalopathy.

### 6H2 modulated leukocyte infiltration and attenuated A-FABP accumulation in microglia and neurons in LPS-induced sepsis

**Fig. 5:**
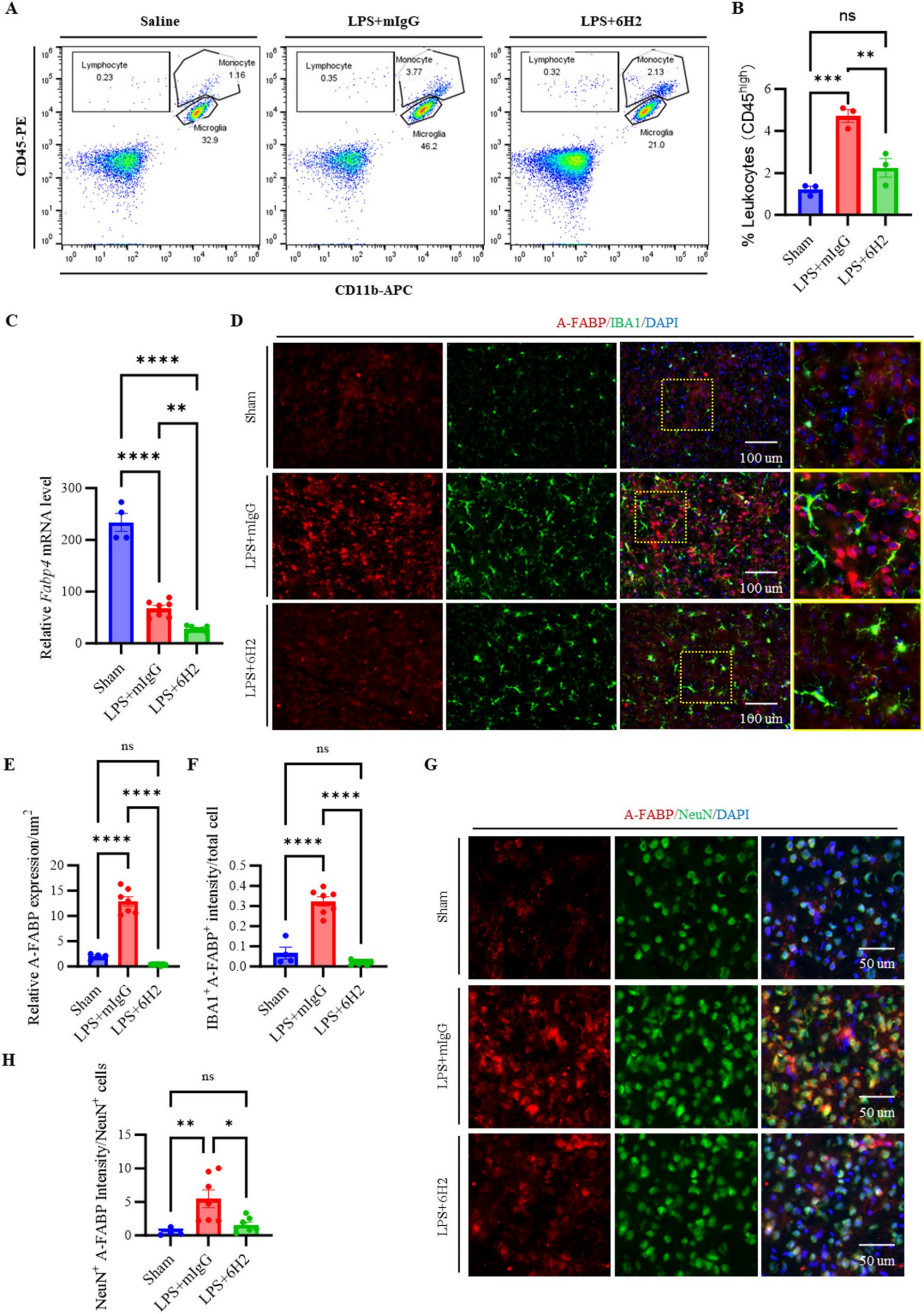
The A-FABP mAb 6H2 modulates A-FABP-mediated neuroinflammation through multiple mechanisms. **A**-**B** Flow cytometry analysis revealed that 6H2 reduced LPS-induced CD45^high^ leukocyte infiltration into the brain. Lymphocyte: CD45^high^CD11b^−^, microglia: CD45^low^CD11b^+^, monocyte: CD45^high^CD11b^+^. **C** RT‒qPCR revealed *Fabp4* mRNA level in the brain. **D**-**F** Co-immunofluorescence staining of A-FABP and IBA1 in the hippocampus. **D** Representative images showed distribution of A-FABP and IBA1. **E** Quantification of A-FABP fluorescence intensity. **F** Quantification of A-FABP amount in the IBA^+^ cells (IBA^+^A-FABP^+^) normalized by total cell. Scale bars: 100 μm. **G**-**H** Co-immunofluorescence staining of A-FABP and NeuN in the hippocampus. **G** Representative images showed distribution of A-FABP and NeuN. **H** Quantification of A-FABP amount in the NeuN^+^ cells (NeuN^+^A-FABP^+^) normalized by NeuN^+^ cells. Scale bars: 50 μm. Data were from animals surviving to the 24-hour endpoint (Sham, *n* = 4; LPS + mIgG, *n* = 7; LPS + 6H2, *n* = 7). The data were presented as the means ± SEM. Statistical significance was determined by one-way ANOVA with Tukey’s post hoc test (*p < 0.05, **p < 0.01, ***p < 0.001, ****p < 0.0001; ns, not significant)

We next investigated whether 6H2 treatment influences A-FABP levels in the brain upon LPS stimulation. First, we conducted FACS to evaluate whether 6H2 affects the infiltration of immune cells in the blood into the brain parenchyma. The Sham group exhibited negligible leukocyte (lymphocyte and monocyte, CD45^high^) infiltration due to the absence of inflammatory stimulation; the LPS + mIgG group exhibited significant leukocyte recruitment (Fig. 5A and B). The LPS + 6H2 group displayed reduced leukocyte infiltration, suggesting that the drug 6H2 mitigated LPS-driven inflammation and BBB disruption.

Next, we assessed *Fabp4* mRNA expression in the brain via RT‒qPCR, followed by quantitative analysis. First, we measured *Fabp4* mRNA expression levels in brain tissue via RT-qPCR. Quantitative RT‒PCR analysis revealed that the Sham group presented the highest expression levels of *Fabp4* mRNA, reflecting baseline expression under normal physiological conditions (Fig. 5C). In contrast, the LPS + mIgG group presented a marked decrease in *Fabp4* expression compared with the sham group, whereas the LPS + 6H2 group presented a further reduction in *Fabp4* mRNA levels. These findings suggest that LPS stimulation downregulates *Fabp4* transcription in the brain and that 6H2 treatment enhances this effect, highlighting its efficacy in modulating *Fabp4* levels in response to LPS-induced inflammation.

To investigate A-FABP protein localization in the brain parenchyma under neuroinflammatory conditions, we performed co-immunofluorescence staining for A-FABP with either IBA1 or NeuN in hippocampal brain tissue. In the LPS + mIgG group, we observed pronounced A-FABP immunoreactivity in both NeuN^+^ neurons (Fig. 5G-H) and IBA1^+^ microglia (Fig. 5D-F), indicating significant A-FABP protein accumulation despite the observed downregulation of *Fabp4* mRNA. This discrepancy suggests that the accumulated A-FABP protein may originate from the peripheral circulation, crossing the compromised BBB under inflammatory conditions and subsequently interacting with neuronal and microglia. In contrast, the LPS + 6H2 group presented attenuated A-FABP staining intensity in both cell types, suggesting that 6H2 treatment reduces A-FABP accumulation. Minimal A-FABP immunoreactivity was detected in the sham group, which served as a baseline control (Fig. 5D-E). To quantify A-FABP accumulation in microglia, we analyzed the integrated density (IntDen) of A-FABP within IBA1^+^ cells, which was normalized to the total cell count (IBA1^+^ A-FABP^+^ IntDen/Total Cell). The LPS + mIgG group presented a small portion of A-FABP^+^ intensity in the IBA1^+^ cells, confirming low level of A-FABP deposition in microglia. Conversely, the LPS + 6H2 group presented a further lower A-FABP^+^IBA1^+^ intensity, confirming that 6H2 treatment mitigated A-FABP accumulation in microglia (Fig. 5F). Similarly, co-staining for A-FABP and NeuN revealed robust A-FABP immunoreactivity in the neurons of the LPS + mIgG-treated mice (NeuN^+^A-FABP^+^ intensity), whereas those in the LPS + 6H2 group decreased significantly (Fig. 5G). Quantification of A-FABP^+^NeuN^+^ intensity further supported these observations, demonstrating that 6H2 treatment significantly reduced A-FABP accumulation in neurons (Fig. 5H).

These findings collectively demonstrate that while LPS-induced neuroinflammation suppresses A-FABP mRNA expression, it promotes A-FABP protein accumulation mostly in the neurons, likely through the infiltration of circulating A-FABP across the disrupted BBB. Notably, 6H2 treatment not only further reduced A-FABP transcription but also attenuated A-FABP protein deposition in these cell populations. This dual regulatory effect suggests that the modulation of A-FABP levels, both at the transcriptional and protein accumulation levels, contributes to the anti-inflammatory and neuroprotective properties of 6H2.

### A-FABP synergizes with LPS to drive late-stage neuronal apoptosis in vitro

Our study revealed an intriguing phenomenon: while LPS stimulation dramatically downregulated *Fabp4* mRNA expression in the brain, A-FABP protein accumulated prominently in neurons. This discrepancy suggests that neuronal A-FABP accumulation may not stem from endogenous production but rather from the uptake of circulating A-FABP, which crosses the compromised BBB during systemic inflammation. To investigate this hypothesis, we examined the direct effects of A-FABP on neuronal cells via the HT22 hippocampal neuronal line and evaluated its effects on A-FABP expression, protein accumulation, cell viability, and apoptosis in the presence or absence of LPS.

**Fig. 6:**
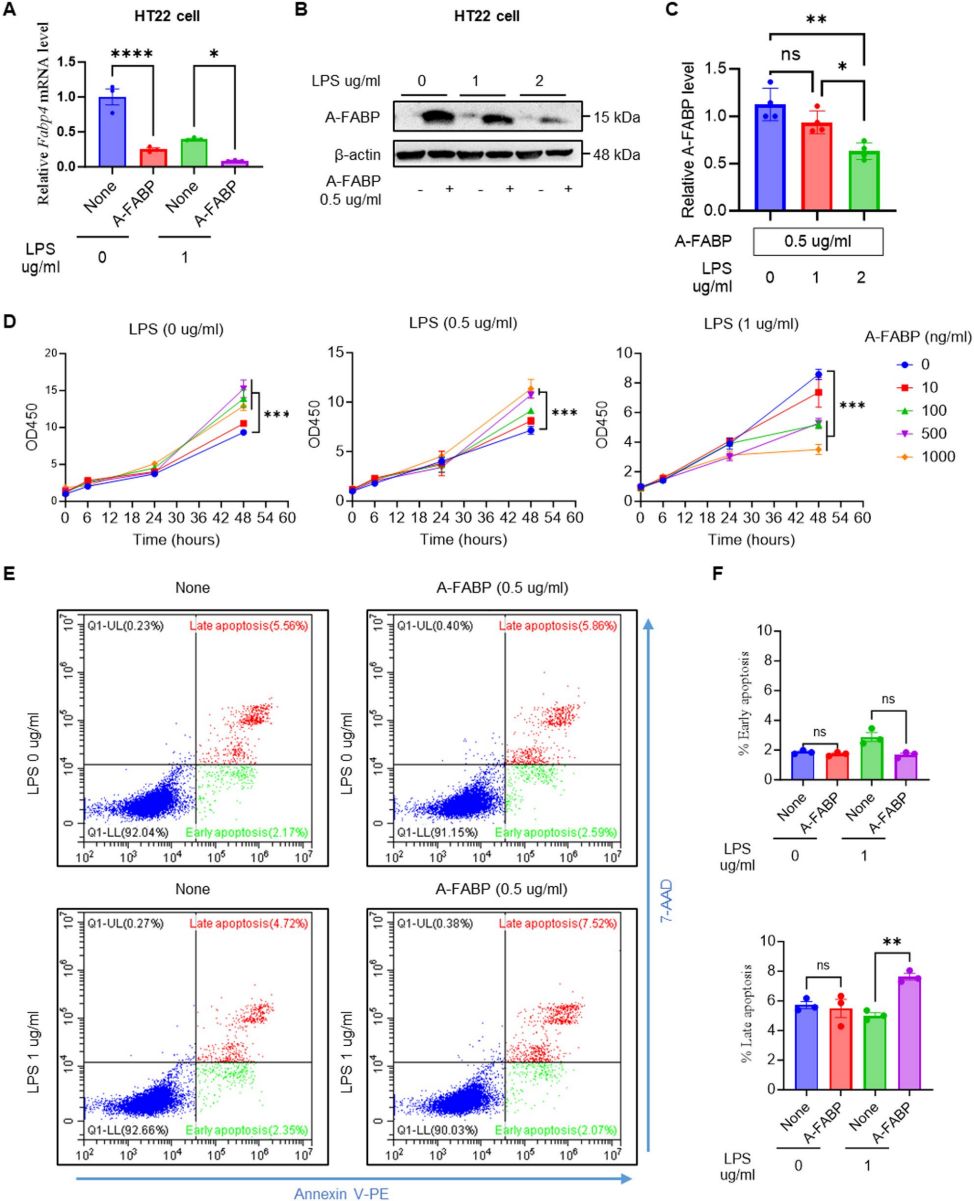
A-FABP modulates A-FABP expression and enhances LPS-induced cytotoxicity in HT22 neurons. HT22 cells were treated with or without recombinant A-FABP (0.5 µg/ml) in the presence or absence of LPS (1 µg/ml or 2 µg/ml) for 24 h. **A***Fabp4* mRNA expression levels were measured by RT-qPCR. **B** Representative Western blot of A-FABP protein levels. **C** Quantification of A-FABP protein levels normalized to β-actin. **D** Cell viability (OD450) was measured over time via a CCK-8 assay in cells treated with various concentrations of recombinant A-FABP with or without LPS. **E** Representative flow cytometry plots showing Annexin V‒PE and 7-AAD staining of HT22 cells. **F** Quantification of early apoptosis (annexin V^+^/7-AAD^−^) and late apoptosis (annexin V^+^/7-AAD^+^). The data were presented as the means ± SEM. Statistical significance was determined by one-way ANOVA with Tukey’s post hoc test (*p < 0.05, **p < 0.01, ***p < 0.001, ****p < 0.0001; ns, not significant)

Our quantitative PCR analysis revealed that recombinant A-FABP significantly suppressed *Fabp4* mRNA expression in HT22 cells at baseline (0 µg/ml LPS). Upon stimulation with LPS (1 µg/ml), the endogenous *Fabp4* mRNA was already downregulated, and exogenous A-FABP further decreased its transcriptional level **(**Fig. 6A**).** These results suggest that extracellular A-FABP contributes to a feedback mechanism that reduces its gene expression. Western blotting analysis revealed that HT22 cells did not express A-FABP, at least at low levels, while A-FABP administration led to a robust A-FABP band, independent of the presence of LPS **(**Fig. 6B**)**. Quantification confirmed that A-FABP protein levels decreased with increasing LPS concentration and that LPS itself could not induce A-FABP expression in neurons **(**Fig. 6C**)**. Complementing these neuronal findings, we also determined whether microglia could serve as an alternative source of A-FABP. We performed parallel experiments using the BV2 microglial cell line. BV2 cells neither took up exogenous A-FABP nor exhibited induced endogenous A-FABP expression upon LPS stimulation (Fig. S1). Collectively, these findings demonstrate that HT22 hippocampal neurons possess minimal endogenous A-FABP production but rapidly accumulate the protein upon exposure to exogenous A-FABP, irrespective of LPS. This, together with the lack of A-FABP induction or uptake in BV2 microglia, strongly supports our hypothesis that neuronal A-FABP accumulation observed in vivo originates primarily from the uptake of circulating A-FABP rather than from *de novo* synthesis within the central nervous system.

To determine whether internalized A-FABP directly affects neuronal viability, we treated HT22 cells with increasing concentrations of recombinant A-FABP (10–1000 ng/ml) in the presence or absence of LPS. In contrast, low concentrations of A-FABP alone did not improve cell viability (Fig. 6D). Instead, higher A-FABP doses (≥ 100 ng/ml) with or without low concentration of LPS (0.5 µg/ml) promoted HT22 survival in a dose-dependent manner, suggesting a neuroprotective effect of exogenous A-FABP under basal conditions and low level LPS stimulation. Strikingly, when A-FABP was combined with high concentration of LPS (1 µg/ml), A-FABP synergistically amplified LPS-induced cytotoxicity. This effect was most pronounced at higher A-FABP concentrations (500–1000 ng/ml), where cell viability decreased significantly compared with that of the cells treated with LPS alone. Time-course experiments further revealed that prolonged exposure (48 h) to A-FABP and LPS exacerbated neuronal death, highlighting a time- and dose-dependent neurotoxic synergy between these two factors (Fig. 6D).

To investigate the synergistic neurotoxic effects of A-FABP and LPS, we quantified the degree of apoptosis in HT22 neurons via Annexin V-PE/7-AAD staining and flow cytometry (Fig. 6E). While neither A-FABP (0.5 µg/ml) nor LPS (1 µg/ml) alone significantly increased apoptosis compared with that of the controls, their combination resulted in a marked increase in late apoptotic cells (Annexin V^+^/7-AAD^+^; *p* < 0.01 vs. LPS alone). Notably, early apoptosis (Annexin V^+^/7-AAD^−^) remained unaffected across all treatment groups (Fig. 6F). The selective increase in late apoptosis suggested that A-FABP exacerbates terminal cell death pathways after LPS primes for neuronal damage.

Collectively, our findings demonstrated a coordinated mechanism of neuronal damage involving A-FABP and LPS. This synergistic interaction provided a mechanistic explanation for the exacerbated neuronal injury observed under inflammatory conditions, where both systemic A-FABP elevation and LPS exposure occur concurrently.

### A-FABP potentiates LPS-induced neuronal apoptosis in vivo

**Fig. 7:**
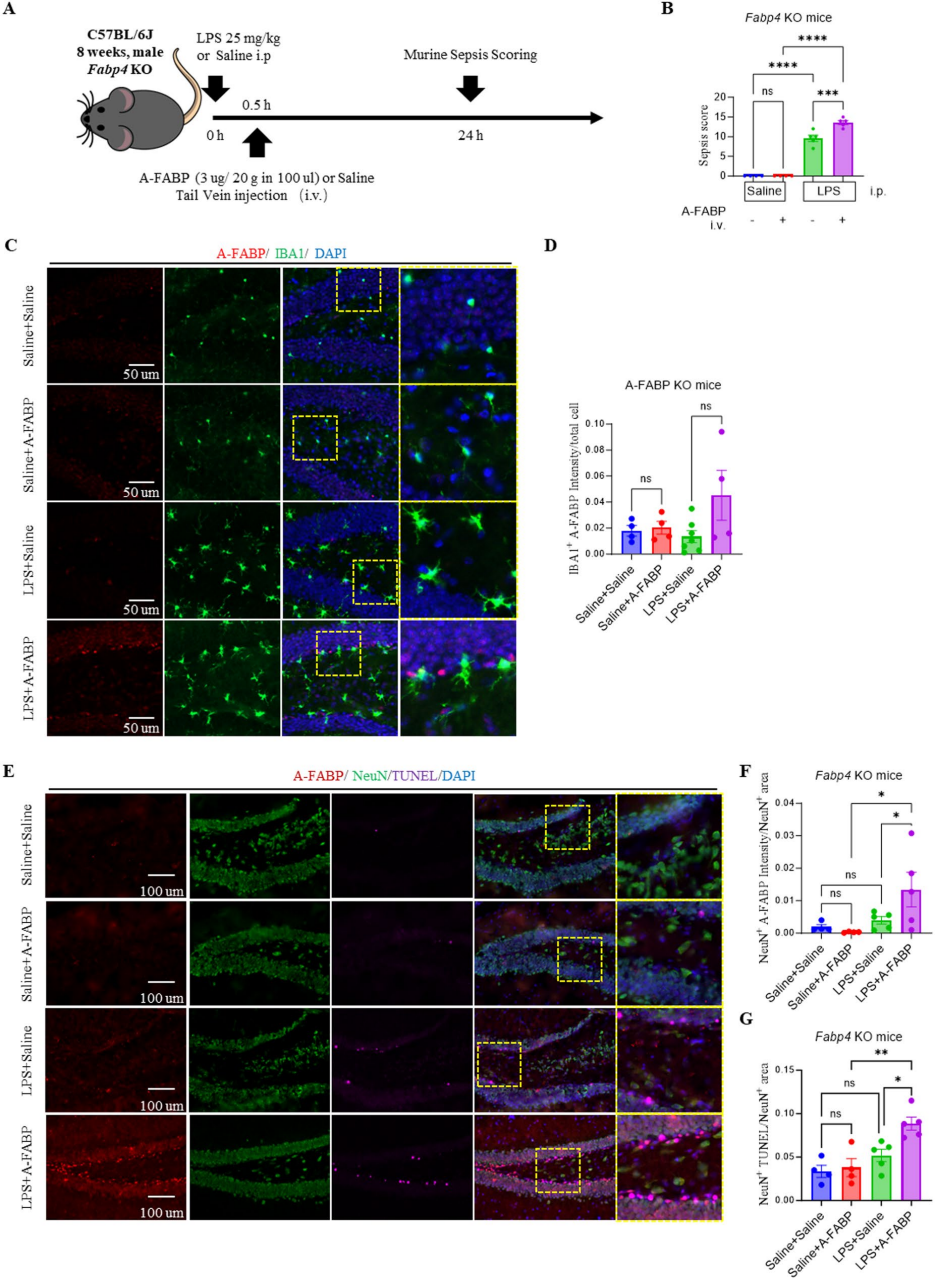
Synergistic effect of A-FABP and LPS on neuronal apoptosis in *Fabp4* knockout mice. **A** Schematic of the experimental design in *Fabp4* KO mice. **B** Murine Sepsis Score (MSS) assessed at 24 h post-treatment. **C** Representative immunofluorescence images of hippocampal sections stained for microglia (IBA1, green) and A-FABP (red). Scale bar: 50 μm. **D** Quantification of A-FABP intensity located in the IBA^+^ cells normalized with total cell number (IBA1^+^ A-FABP intensity/total cell) in the hippocampal CA1 region. **E** Representative immunofluorescence images of hippocampal sections stained for neurons (NeuN, green), A-FABP (red) and apoptotic cells (TUNEL, magenta). Scale bar: 100 μm. **F** Quantification of A-FABP intensity located in the NeuN^+^ cells normalized with NeuN area (NeuN^+^ A-FABP intensity/NeuN^+^ area) in the hippocampal CA1 region. **G** Quantification of apoptotic NeuN⁺ cells normalized by NeuN area (NeuN^+^ TUNEL/NeuN^+^ area) in the hippocampal CA1 region. Data were presented as mean ± SEM. Statistical significance was determined by one-way ANOVA with Tukey’s post-hoc test (*p < 0.05, **p < 0.01, ***p < 0.001, ****p < 0.0001; ns, not significant)

To validate our in vitro observations of the synergistic neurotoxic effects of A-FABP and LPS, we performed complementary in vivo experiments in *Fabp4* knockout (KO) mice. 8-week-old male *Fabp4* KO mice were intraperitoneally injected with either LPS (25 mg/kg, i.p.) to induce endotoxemia or saline as a control. 30 min later, the mice received an intravenous injection of either recombinant A-FABP (3 µg) or saline through the tail vein (Fig. 7A).

Assessment of sepsis severity revealed that saline-treated controls (Saline + Saline and Saline + A-FABP) maintained low clinical scores. LPS challenge induced severe systemic inflammation, significantly elevating sepsis scores in both LPS-treated groups, confirming that A-FABP deletion did not prevent sepsis onset. Notably, mice receiving both LPS and exogenous A-FABP (LPS + A-FABP) exhibited even higher sepsis scores than those treated with LPS alone (LPS + Saline) (Fig. 7B), indicating that circulating A-FABP actively worsens disease severity.

We next examined A-FABP distribution in the brain. Co-immunostaining for the microglial marker IBA1 and A-FABP showed minimal A-FABP signal in microglia across all groups, including the LPS + A-FABP group (Fig. 7C, D), suggesting that microglia are not a primary site of A-FABP accumulation under these conditions. In contrast, robust A-FABP immunoreactivity was detected specifically in hippocampal neurons of LPS + A-FABP-treated mice (Fig. 7E, F). This pattern demonstrates that LPS-induced BBB disruption permits circulating A-FABP to enter the brain parenchyma, where it is subsequently internalized by neurons.

Consistent with this localization, neuronal apoptosis was markedly exacerbated in the LPS + A-FABP group. While saline controls showed healthy neurons (NeuN⁺) with minimal apoptosis (TUNEL⁺), LPS alone increased neuronal apoptosis. Co-administration of LPS and A-FABP resulted in a significantly greater proportion of TUNEL⁺ neurons (NeuN⁺/TUNEL⁺ double-labeled cells) compared to LPS alone (Fig. 7G), confirming a synergistic pro-apoptotic effect in vivo.

Together, these data demonstrate that during systemic inflammation, circulating A-FABP crosses the compromised BBB, accumulates in hippocampal neurons, and synergistically amplifies LPS-induced neuronal apoptosis. This establishes A-FABP as an active mediator of neurotoxic injury and a promising therapeutic target for sepsis-associated neurodegeneration.

## Discussion

Our study elucidates a novel mechanism in sepsis-associated encephalopathy (SAE), identifying circulating adipocyte fatty acid-binding protein (A-FABP) as a key pathogenic mediator that bridges systemic inflammation with central nervous system (CNS) injury. These results align with previous studies showing that systemic inflammation disrupts the BBB, allowing harmful substances to infiltrate the central nervous system (CNS) [38–40]. We demonstrate that neutralization of A-FABP with the monoclonal antibody 6H2 confers significant neuroprotection in a murine endotoxemia model, primarily through the preservation of blood-brain barrier (BBB) integrity, attenuation of neuroinflammation, and reduction of neuronal apoptosis. These findings position A-FABP not only as a disease biomarker but as an actionable therapeutic target for SAE and related neuroinflammatory conditions.

The efficacy of 6H2 treatment stemmed from its multi-faceted actions. It markedly reduced the extravasation of Evans Blue and endogenous IgG into the brain parenchyma, indicating a stabilization of the BBB, a critical initial step in preventing neuroinflammation. This effect is likely attributable to the antibody’s capacity to neutralize circulating A-FABP, thereby limiting its pathological influx across a vulnerable BBB, a process corroborated by our imaging studies in *Fabp4* knockout mice. Concurrently, 6H2 treatment dampened the CNS inflammatory cascade, as shown by decreased levels of pro-inflammatory cytokines (*Tnf*, *Il1b*, *Il6*, *Il17a*, and *Ccl2*) and a reversion of microglia from an activated, amoeboid morphology toward a homeostatic, ramified state. This suggests that A-FABP neutralization can actively promote resolution of glial activation.

A pivotal finding was the discordance between *Fabp4* gene expression and protein accumulation in the LPS-challenged brain. While *Fabp4* mRNA was downregulated, we observed robust A-FABP protein deposition in hippocampal neurons. Our integrated in vitro and in vivo data resolve this paradox by supporting a model of peripheral origin and cellular uptake. HT22 neuronal cells, which exhibit minimal endogenous A-FABP, readily internalized exogenous protein. In contrast, BV2 microglia neither produced significant *de novo* A-FABP nor took up the exogenous protein under these conditions. Thus, the A-FABP accumulating in the brain during endotoxemia appears predominantly blood-derived, crossing the compromised BBB and being internalized by receptive neurons.

This pathway has direct neurotoxic consequences. We identified a potent synergistic interaction between A-FABP and LPS in driving neuronal apoptosis. In vitro, A-FABP co-treatment with LPS specifically exacerbated late-stage apoptotic pathways. In vivo, administration of exogenous A-FABP to LPS-challenged mice significantly worsened hippocampal neuronal apoptosis and clinical severity compared to LPS alone. This reveals a “two-hit” mechanism: LPS induces BBB dysfunction and primes cellular stress, while circulating A-FABP infiltrates the CNS to execute and amplify apoptotic signaling. The therapeutic action of 6H2 lies in intercepting this second hit.

Our study also provides initial insight into the therapeutic window of A-FABP neutralization. While early administration (30-min post-LPS) was most effective, delayed treatment (3-hours post-LPS) still yielded significant neuroprotection, particularly in preserving BBB integrity and reducing neuronal apoptosis, underscoring its potential clinical relevance.

Several important questions arise from this work. Future studies should delineate the precise molecular mechanisms of neuronal A-FABP uptake and the intracellular pathways through which it synergizes with LPS to induce apoptosis. Furthermore, investigations in more chronic sepsis models and assessments of long-term cognitive outcomes after A-FABP neutralization will be crucial for validating its full therapeutic promise.

In conclusion, this work establishes circulating A-FABP as a critical pathogenic signal in SAE, exacerbating brain injury via synergistic potentiation of LPS-induced neurotoxicity. The neutralizing antibody 6H2 effectively targets this pathway, preserving the neurovascular unit and improving outcomes. These findings offer a novel and translatable strategy for mitigating brain dysfunction in sepsis and potentially other conditions characterized by systemic inflammation coupled with BBB compromise.

## Supplementary Information

Supplementary Material 1.

Supplementary Material 2.

Supplementary Material 3.

Supplementary Material 4.

## Data Availability

No datasets were generated or analyzed during the current study.
