# Supplementary material for: Circulating adipocyte fatty acid-binding protein exacerbates LPS-induced neurotoxicity by crossing the disrupted blood–brain barrier and promoting neuronal apoptosis
Source: Cell Commun Signal. 2026 Jan 23;24:119. doi: 10.1186/s12964-026-02680-y (PMC12910782; doi:10.1186/s12964-026-02680-y)
Supplement: Supplementary file 1 — Supplementary Material 1. [file 12964_2026_2680_MOESM1_ESM.docx]

**Table S1. Antibodies**

| **Target antigen** | **Vendor or Source** | **Catalog #** | **Working concentration** |
| --- | --- | --- | --- |
| **Primary Antibodies** | | | |
| CD31 | Millipore | MAB1398Z | 1:1000 (IF) |
| Iba1 | ABclonal | A19776 | 1:100 (IF) |
| Anti-m/rFABP4 | R & D System | IOGO719051 | 1:200 (IF) |
| **Secondary Antibodies** | | | |
| HRP-anti-mouse IgG | Cell Signaling Technology | 7076 | 1:1000 (WB) |
| anti-GFAP | Thermo Fisher Scientific, USA) | cat. #PA1-10004, | (1:1000) (IF) |
| anti-NeuN | Cell Signaling Technology, USA | cat. #94403, | (1:500) (IF) |
| TUNEL (YSFluorTM 640) | YEASON | 40308ES20 |  |
| Alexa Fluor 488 Donkey Anti-Mouse IgG | Jackson ImmunoResearch | 715-545-150 | 1:500 (IF) |
| Alexa Fluor 488 Donkey Anti-Rabbit IgG | Jackson ImmunoResearch | 711-545-152 | 1:500 (IF) |
| Alexa Fluor 647 goat anti-rabbit IgG | Jackson ImmunoResearch | 711-605-152 | 1:500 (IF) |
| Alexa Fluor 594 goat anti-mouse IgG | Jackson ImmunoResearch | 715-585-150 | 1:500 (IF) |
| HRP-Conjugated Anti-Goat IgG | Beyotime | A0181 | 1:500 (IF) |

**Table S2:** **Primer sequences used for analyzing the transcript levels of Proinflammatory cytokines**

| SN | Primer(s) Name (Forward and Reverse) | Sequence |
| --- | --- | --- |
| 1 | mTnf-a (f) | CCCTCACACTCAGATCATCTTCT |
| 2 | mTnf-a (r) | GCTACGACGTGGGCTACAG |
| 3 | mIL-1β (f) | GCAACTGTTCCTGAACTCAACT |
| 4 | mIL-1β (r) | ATCTTTTGGGGTCCGTCAACT |
| 5 | mIL-6 (f) | CTGCAAGAGACTTCCATCCAG |
| 6 | mIL-6 (r) | AGTGGTATAGACAGGTCTGTTGG |
| 7 | mIL-17a (f) | TTTAACTCCCTTGGCGCAAAA |
| 8 | mIL-17a (f) | CTTTCCCTCCGCATTGACAC |
| 9 | mCcl2 (f) | TTAAAAACCTGGATCGGAACCAA |
| 10 | mCcl2 (r) | GCATTAGCTTCAGATTTACGGGT |
| 11 | mAfabp44 (f) | CGATGAAATCACCGCAGACG |
| 12 | mAfabp4 (r) | CCAGCTTGTCACCATCTCGT |
| 13 | mβ-actin (f) | GGCTGTATTCCCCTCCATCG |
| 14 | mβ-actin (r) | CCAGTTGGTAACAATGCCATGT |

**Table S3. Murine Sepsis Score (MSS) to assess the severity of disease in an experimental model of sepsis induced by LPS**

| **Variable** | **Score and description** |
| --- | --- |

**Appearance** 0. Coat is smooth

1. Patches of hair piloerected
2. The majority of the back is piloerected
3. Piloerection may or may not be present, the mouse appears “puffy”
4. Piloerection may or may not be present, mouse appears emaciated

**Level of consciousness** 0. Mouse is active

1. The mouse is active but avoids standing upright
2. Mouse activity is noticeably slowed. The mouse is still ambulant.
3. Activity is impaired. Mouse only moves when provoked, movements have a tremor
4. Activity severely impaired. Mouse remains stationary when provoked, with possible tremor

**Activity** 0. Normal amount of activity. Mouse is any of: eating, drinking, climbing, running, fighting

1. Slightly suppressed activity. The mouse is moving around the bottom of the cage
2. Suppressed activity. The mouse is stationary with occasional investigative movements
3. No activity. Mouse is stationary
4. No activity. Mouse experiencing tremors, particularly in the hind legs

**Response to stimulus**  0. The Mouse responds immediately to auditory stimulus or touch

1. Slow or no response to auditory stimulus; strong response to touch (moves to escape)
2. No response to auditory stimulus; moderate response to touch (moves a few steps)
3. No response to auditory stimulus; mild response to touch (no locomotion)
4. No response to auditory stimulus. Little or no response to touch. It cannot right itself if pushed over

**Eyes**  0. Open

1. Eyes not fully open, possibly with secretions
2. Eyes at least half closed, possibly with secretions
3. Eyes half closed or more, possibly with secretions
4. Eyes closed or milky

**Respiration rate** 0. Normal, rapid mouse respiration

1. Slightly decreased respiration (rate not quantifiable by eye)
2. Moderately reduced respiration (rate at the upper range of quantifying by eye)
3. Severely reduced respiration (rate easily countable by eye, 0.5 s between breaths)
4. Extremely reduced respiration (>1 s between breaths)

**Respiration quality** 0. Normal

1. Brief periods of labored breathing
2. Laboured, no gasping
3. Laboured with intermittent gasps
4. Gasping
