# Supplementary figures and images for: Circulating adipocyte fatty acid-binding protein exacerbates LPS-induced neurotoxicity by crossing the disrupted blood–brain barrier and promoting neuronal apoptosis
Source: Cell Commun Signal. 2026 Jan 23;24:119. doi: 10.1186/s12964-026-02680-y (PMC12910782; doi:10.1186/s12964-026-02680-y)

Fig. 2B

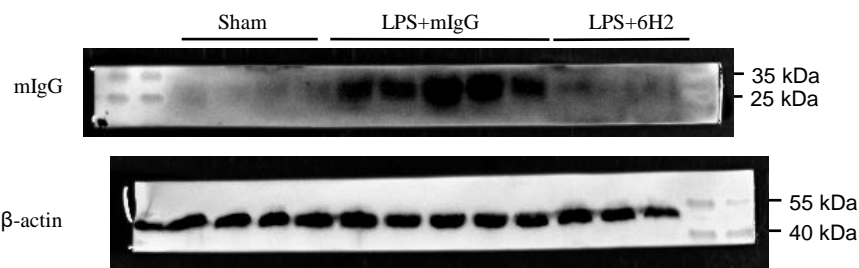

Fig. 6B

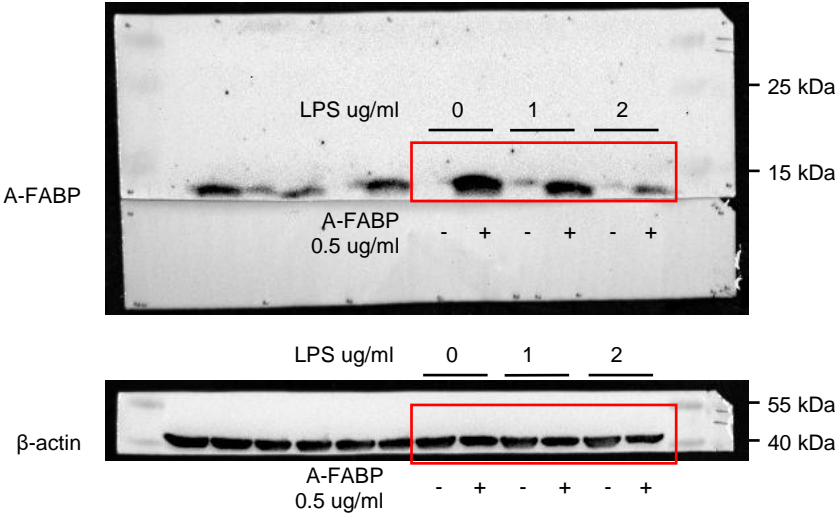

Supplement: Supplementary file 2 — Supplementary Material 2. [file 12964_2026_2680_MOESM2_ESM.pdf]
