# Supplementary material for: Circulating adipocyte fatty acid-binding protein exacerbates LPS-induced neurotoxicity by crossing the disrupted blood–brain barrier and promoting neuronal apoptosis
Source: Cell Commun Signal. 2026 Jan 23;24:119. doi: 10.1186/s12964-026-02680-y (PMC12910782; doi:10.1186/s12964-026-02680-y)
Supplement: Supplementary file 3 — Supplementary Material 3. [file 12964_2026_2680_MOESM3_ESM.docx]

**Supplemental Material for:**

**Circulating Adipocyte Fatty Acid-Binding Protein Exacerbates LPS-Induced Neurotoxicity by Crossing the Disrupted Blood–Brain Barrier and Promoting Neuronal Apoptosis**

Muhammad Mustapha Ibrahim^1,2,8^, Chunyan Li^1,3,8^, Linhui Qiu^1^, Yue Hu^4,5^, Aimin Xu^4,5^, Shilun Yang^1*^, Junlei Chang^1,6,7*^, Cheng Fang^1*^

^1^State Key Laboratory of Biomedical Imaging Science and System, Guangdong-Hong Kong Joint Laboratory for Metabolic Medicine, Institute of Biomedicine and Biotechnology, Shenzhen Institute of Advanced Technology, Chinese Academy of Sciences, Shenzhen, Guangdong, China.

^2^University of Chinese Academy of Sciences, Beijing, China

^3^Department of Biomedical Engineering, Southern University of Science and Technology, Shenzhen, Guangdong, China

^4^Department of Pharmacology and Pharmacy, LKS Faculty of Medicine, The University of Hong Kong, Pokfulam, Hong Kong, China

^5^State Key Laboratory of Pharmacological Biotechnology, LKS Faculty of Medicine, The University of Hong Kong, Pokfulam, Hong Kong, China

^6^Stroke Center, Department of Neurology, The First Hospital of Jilin University, Changchun, China

^7^Neuroscience Research Center, Department of Neurology, The First Hospital of Jilin University, Changchun, Jilin, China.

^8^These authors contributed equally to this work.

**^*^Correspondence should be addressed to:**

**Cheng Fang, PhD**. Institute of Biomedicine and Biotechnology, Shenzhen Institute of Advanced Technology, Chinese Academy of Sciences. Xueyuan Avenue 1068, Nanshan, Shenzhen 518055, Guangdong, China. E-mail: [cheng.fang@siat.ac.cn](mailto:cheng.fang@siat.ac.cn). ORCID number: 0009-0003-3545-0116.

**Junlei Chang, PhD**. Institute of Biomedicine and Biotechnology, Shenzhen Institute of Advanced Technology, Chinese Academy of Sciences. Xueyuan Avenue 1068, Nanshan, Shenzhen 518055, Guangdong, China. E-mail: [jl.chang@siat.ac.cn](mailto:jl.chang@siat.ac.cn). ORCID number: 0000-0002-0319-9022.

**Shilun Yang, PhD**. Institute of Biomedicine and Biotechnology, Shenzhen Institute of Advanced Technology, Chinese Academy of Sciences. Xueyuan Avenue 1068, Nanshan, Shenzhen 518055, Guangdong, China. E-mail: [sl.yang@siat.ac.cn](mailto:sl.yang@siat.ac.cn). ORCID number: 0000-0003-2734-3698.

**Figure S1-S2**

**
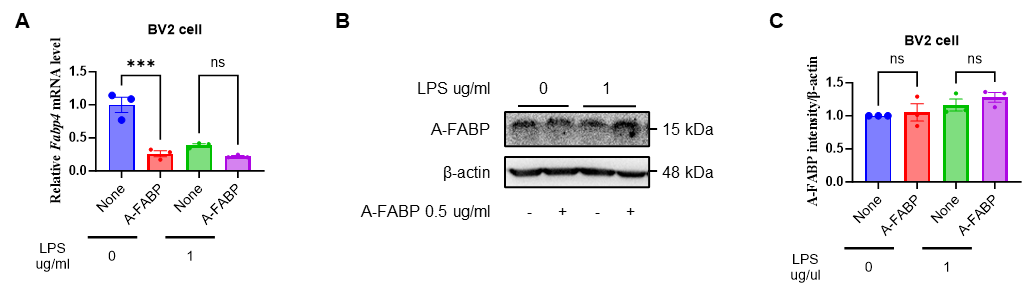
**

**Figure S1.** BV2 microglial cells did not take up exogenous A-FABP protein.

BV2 cells were treated with or without recombinant A-FABP (0.5 μg/ml) in the presence or absence of LPS (1 μg/ml) for 24 hours. (**A**) *Fabp4* mRNA expression levels were measured by RT-qPCR. (**B**) Representative Western blot of A-FABP protein levels. (**C**) Quantification of A-FABP protein levels normalized to β-actin. The data were presented as the mean ± SEM. Statistical significance was determined by one-way ANOVA with Tukey’s post hoc test (****p* < 0.001; ns, not significant).

**
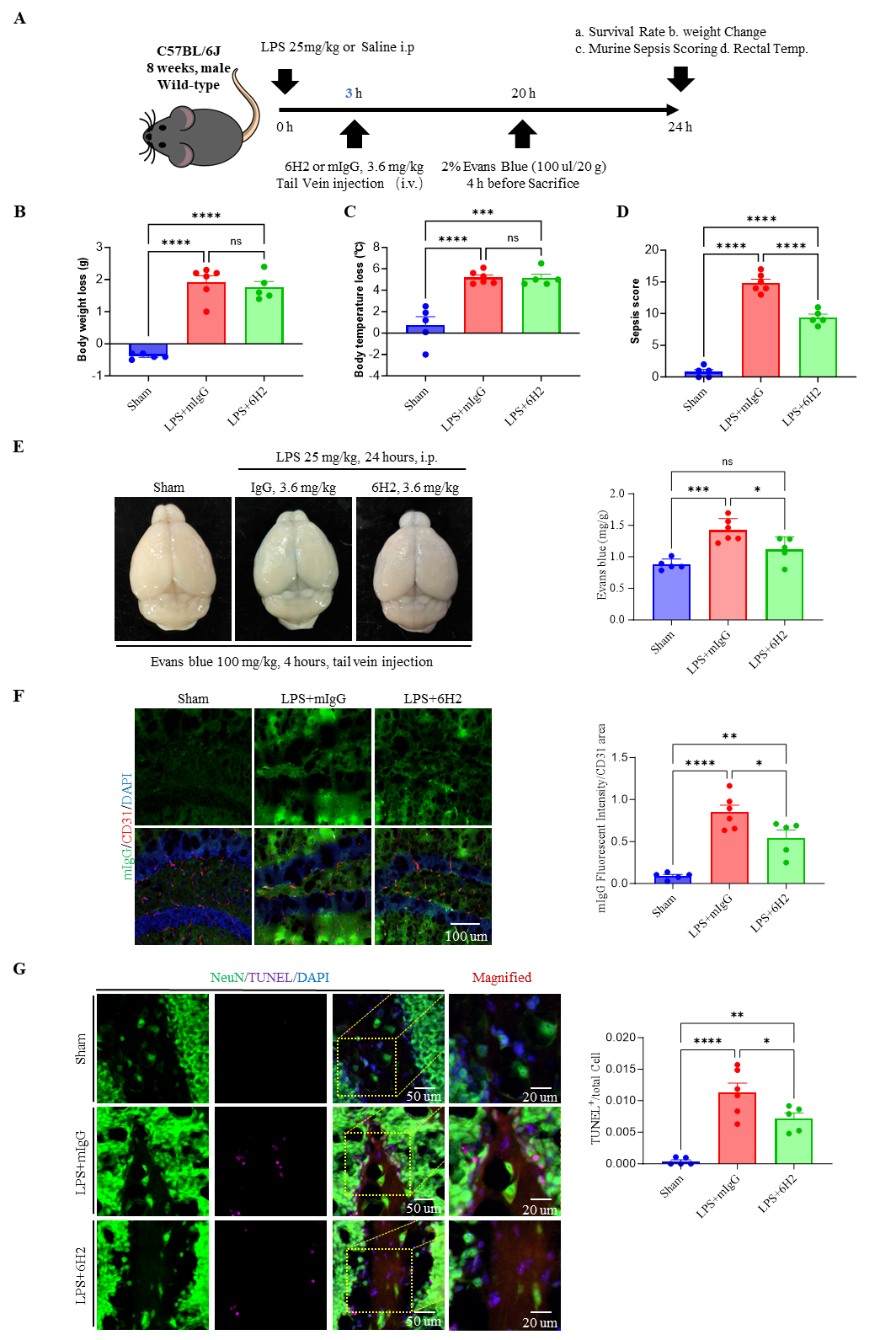
**

**Figure S2.** Neuroprotective efficacy of 6H2 when administered 3 hours post-LPS.

(**A**) Schematic of the delayed-treatment protocol. **(B, C**) LPS induced significant weight loss and hypothermia, which were not reversed by delayed 6H2 treatment. (**D**) 6H2 treatment significantly reduced the Murine Sepsis Score. (**E, F**) 6H2 significantly attenuated BBB disruption, as shown by reduced Evans Blue extravasation (**E**) and decreased IgG leakage (**F**). (**G**) 6H2 significantly decreased the number of TUNEL-positive apoptotic neurons. Data were from animals surviving to the 24-hour endpoint (Sham, n=5; LPS+mIgG, n=6; LPS+6H2, n=5). The data were presented as the mean ± SEM. Statistical significance was determined by one-way ANOVA with Tukey’s post hoc test (**p* < 0.05, ***p* < 0.01, ****p* < 0.001, *****p* < 0.0001; ns, not significant).

**Table S1. Primer sequences used for RT-qPCR**

| **Gene** | **Forward (5’ to 3’ sequence)** | **Reverse (5’ to 3’ sequence)** |
| --- | --- | --- |
| m*Tnf* | CCCTCACACTCAGATCATCTTCT | GCTACGACGTGGGCTACAG |
| m*Il1b* | GCAACTGTTCCTGAACTCAACT | ATCTTTTGGGGTCCGTCAACT |
| m*Il6* | CTGCAAGAGACTTCCATCCAG | AGTGGTATAGACAGGTCTGTTGG |
| m*Il17a* | TTTAACTCCCTTGGCGCAAAA | CTTTCCCTCCGCATTGACAC |
| m*Ccl2* | TTAAAAACCTGGATCGGAACCAA | GCATTAGCTTCAGATTTACGGGT |
| m*Fabp4* | CGATGAAATCACCGCAGACG | CCAGCTTGTCACCATCTCGT |
| m*b-actin* | GGCTGTATTCCCCTCCATCG | CCAGTTGGTAACAATGCCATGT |

**Table S2. Mouse information in each experiment**

| **Experiment times** | **Mice information** | **Experiments** |
| --- | --- | --- |
| 1^st^ | **Wild type mice**  Sham: 6 mice, no mice died, n=6  mIgG: 8 mice, 3 mice died, n=5  6H2: 7 mice, 1 mouse died, n=6 | Body weight, body temperature, sepsis score, ELISA (for serum A-FABP measurement) |
| 2^nd^ | **Wild type mice**  Sham: 4 mice, no mice died, n=4  mIgG: 8 mice, 2 mice died, n=6  6H2: 7 mice, 1 mouse died, n=6 | Evans blue experiment, IgG staining. |
| 3^nd^ | **Wild type mice**  Sham: 4 mice, no mice died, n=4  mIgG: 10 mice, 3 mice died, n=7  6H2: 9 mice, 2 mice died, n=7 | Western blot, Immunostaining |
| 4^th^ | **Wild type mice**  Sham: 6 mice, no mice died, n=3 (2 mice per group)  mIgG: 8 mice, 3 mice died, n=3 (1-2 mice per group)  6H2: 7 mice, 2 mice died, n=3 (1-2 mice per group) | Flow cytometry analysis of immune cells in the brain |
| 5^th^ | ***Fabp4* KO mice**  Saline+Saline: 4 mice, no mice died, n=4  Saline+A-FABP: 4 mice, no mice died, n=4  LPS+Saline: 6 mice, 1 mouse died, n=5  LPS+A-FABP: 8 mice, 3 mice died, n=5 | Sepsis score, Immunostaining |
| 6^th^ | ***Fabp4* KO mice**  Saline+Saline: 4 mice, no mice died, n=4  Saline+A-FABP: 4 mice, no mice died, n=4  LPS+Saline: 12 mice, 5 mice died, n=7  LPS+A-FABP: 10 mice, 6 mice died, n=4 | Immunostaining |
| 7^th^ | **Wild type mice**  Sham: 5 mice, no mice died, n=5  mIgG: 8 mice, 2 mice died, n=6  6H2: 8 mice, 3 mice died, n=5 | mIgG or 6H2 administered 3 hours after LPS challenge (**Fig. S2**) |
